# Comb-structured triboelectric nanogenerators for multi-directional energy scavenging from human movements

**DOI:** 10.1080/14686996.2019.1630856

**Published:** 2019-07-01

**Authors:** Hee Jae Hwang, Yeonseok Jung, Kyungwho Choi, Dongseob Kim, Jinhyoung Park, Dukhyun Choi

**Affiliations:** a Department of Mechanical Engineering, Kyung Hee University, Yongin, Korea; b New Transportation Innovative Research Center, Korea Railroad Research Institute, Uiwang-si, Korea; c Korea Institute of Industrial Technology (KITECH), Aircraft System Technology Group, Yeongcheon-si, Gyeongbuk-do, Korea; d Korea Institute of Industrial Technology (KITECH), Mechatronics Technology Convergence R&D Group, Daegu-si, Korea

**Keywords:** Triboelectric nanogenerator, comb structure, multi-directional, hand-held pendulum, velocity monitoring, 10 Engineering and Structural materials, 206 Energy conversion / transport / storage / recovery, 208 Sensors and actuators, 307 Kinetics and energy / mass transport

## Abstract

A triboelectric nanogenerator (TENG) is an emerging energy harvesting technology utilizing multi-directional, wasted mechanical energies stemming from vibrations, winds, waves, body movements, etc. In this study, we report a comb-structured TENG (CTENG) capable of effectively scavenging multi-directional motions from human movements, which include walking, jumping, and running. By attaching CTENG to a person’s calf, we obtain a root-mean-square (RMS) power value of 5.28 μW (i.e. 13.12 V and 0.4 μA) for 1 s during mild running action (~5 m/s), which is sufficient for powering 10 light emitting diodes (LEDs). We integrate a CTENG with a simple hand-held pendulum (HHP) system with a natural frequency of 5.5 Hz. The natural frequency and input energy of our HHP system can be easily controlled by changing the holder mass and initial bending displacement, thus producing different output behaviors for the CTENG. Under the optimal HHP-based CTENG system design, the maximum output reaches 116 V at 6.5 μA under 0.1 kg mass and 4 cm bending displacement conditions. The corresponding output energy is 52.7 μJ for an operation time of 10.8 s. Our HHP-CTENG system can sufficiently power 45 LEDs and shows different output performances by varying the driving velocity of a vehicle, thus demonstrating the possibility for a self-powered velocity monitoring system.

## Graphical abstract

**Figure:**
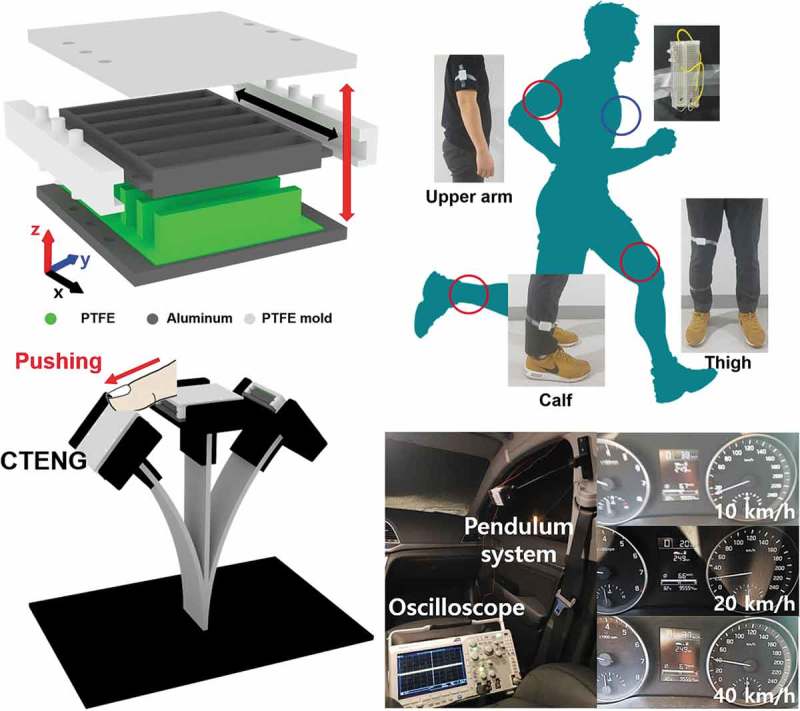

## Introduction

Over the past several decades, given rapid economic development and increasing energy demands, research efforts have been devoted to harvesting ambient environmental energy [1]. Energy demands have increased with the advancement of the Internet of Things (IoT), including sensors and portable devices related to human healthcare, environmental monitoring, industrial manufacturing, and public safety [2,3]. The need for energy harvesting from renewable and clean energy sources, for example human motion, vibration and light, has increased [4–6]. Generally, the electrical power requirement for small electronics and IoT is on the order of the milliwatt (mW) or micro-watt (μW) range, with features such as low cost, mobility, light-weight, and sustainability. Fitting with these concepts is the notion of ‘self-powered’ electronics, which has been proposed and studied here [7–9].

First demonstrated in 2012, the triboelectric nanogenerator (TENG), which combines triboelectrification and electrostatic induction, has attracted significant attention as a promising next-generation energy harvester [10–13]. The main advantage of the TENG is its effectiveness in harvesting mechanical energy or vibrations from our ambient environment at low cost, while retaining scalability to drive low-power, large scale, consumption systems [14]. To date, many researchers have studied the working mechanisms of the TENG and have shown enhancements in TENG output power while utilizing TENGs in various electronic, biomedical, and sensing applications [15–19]. Given that the working mechanism for vertical contact-mode TENGs and sliding-mode TENGs involves repeated contact and separation for energy production, multi-directional motions are difficult to effectively harvest [20]. In previous studies, researchers have designed one-directional TENG devices for scavenging human motion (walking, jumping, and running) [21–23]. However, body movement almost never occurs in a single direction but rather in multiple directions. To effectively scavenge diverse kinetic energies from our environment, it is therefore crucial to design multi-directional TENGs.

In this work, we introduce a multi-axial system utilizing a comb-structure TENG (CTENG) for practically wasting multi-directional mechanical energies such as human body motions and demonstrate their temporary continuous operation via a hand-held pendulum (HHP) system. First, we investigate that the multi-directional operation of the CTENG is influenced by the movable displacement *d_G_* (0, 1, 2, and 2.5 mm) which controls the mobility of the vibrating part (see Figure S1) in z-axis direction. Also, we examine the output performance with motions (walking, jumping, and running) by attaching the CTENG to parts of the human body, including the calf, thigh, and upper arm. Second, we demonstrate that the natural frequency of the HHP system can be controlled by a movable mass (0.1, 0.2, and 0.3 kg) and compare the operation time and efficiency of the HHP with the bending displacement (2, 3, and 4 cm) to design an optimal system. As a result of the research presented here, we have designed an HHP with CTENG capable of supplying electrical energy anywhere, at any time, and as a result of any motion, and further demonstrate a self-powered velocity monitoring system.

## Methods

**Figure 1.**
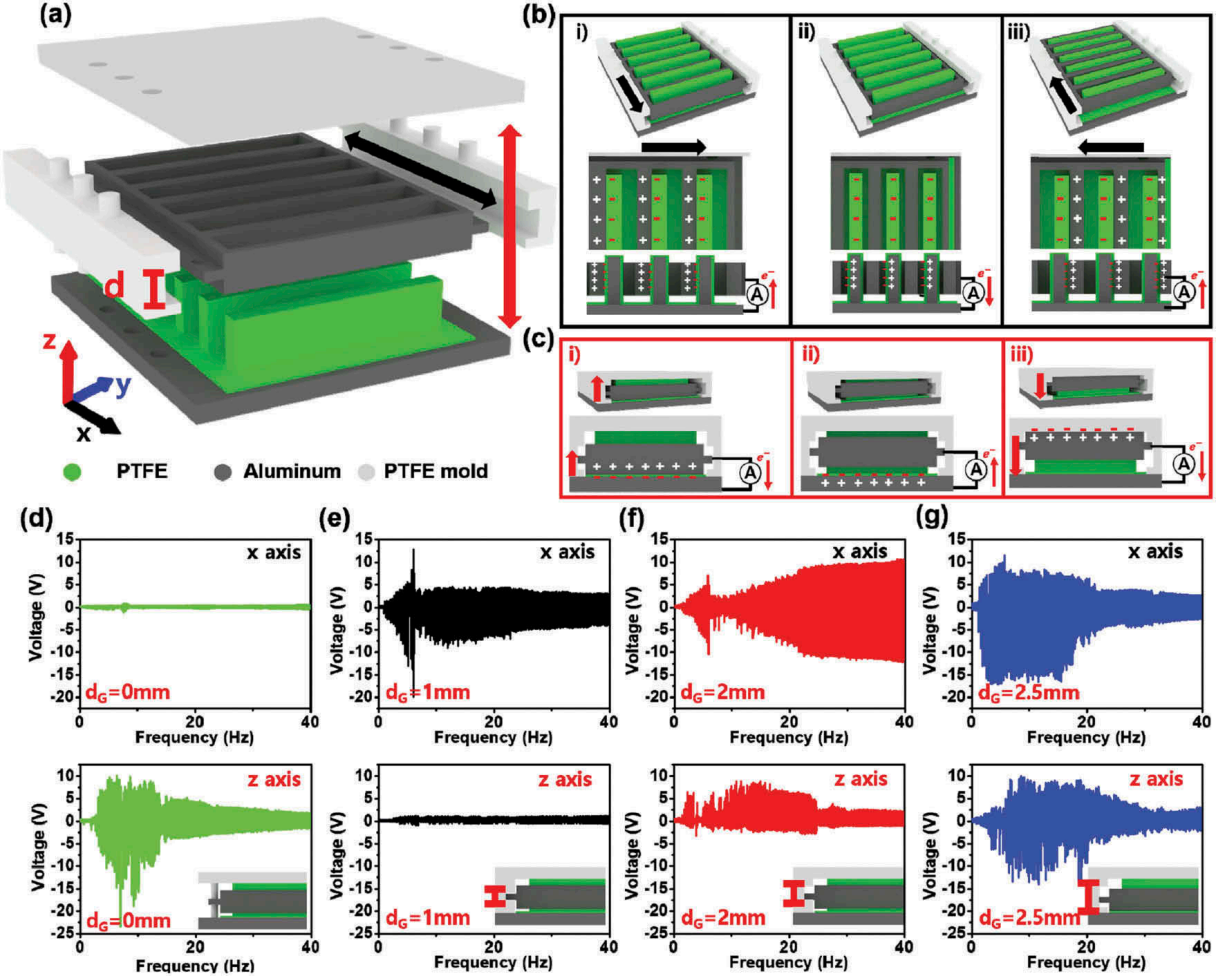
Schematic of the CTENG and output voltage as a function of gap size and frequency sweep. (a) Illustration of CTENG. The working mechanism of the CTENG on the (b) x-axis and (c) z-axis frequency sweep from 0 to 40 Hz. The output performance for d_G_ (d) 0 mm (e) 1 mm (f) 2 mm and (f) 2.5 mm of CTENG during a frequency sweep on the x-axis and z-axis.

A schematic of the CTENG is shown in Figure 1(a) and S1. The device consists of four parts. The first is a top layer consisting of molded polytetrafluoroethylene (PTFE) with the size of 4.5 cm × 3.5 cm (x-y axis), with three circular holes in both the right and left sides. The second part is a middle layer as a vibrating part, comprising an aluminum (Al) mold with the size of 4 cm × 3 cm (x-y axis), with seven rectangular holes with the size of 3 cm × 0.2 cm (x-y axis) each. Side parts are made of molded PTFE with the size of 0.4 cm × 3 cm × 0.6 cm (x-y-z axis) and have a groove to control the movable displacement of the middle vibrating part in the z-direction, where the groove gaps (*d*_G_) were 0, 1, 2, and 2.5 mm. Finally, the bottom layer is molded Al, measuring 4.5 cm × 3.5 cm (x-y axis), containing seven rectangular fins with the size of 3 cm × 0.5 cm × 0.1 cm (x-y-z axis). PTFE tape (80 μm thickness) was attached to the bottom layer as a negative triboelectric material, and a positive triboelectric layer was the middle vibrating part (Al). The vibration test equipment setup consisted of an ET-139 electromagnetic shaker and a PA-138 linear power amplifier (Labworks Inc., USA). To measure the output voltage and current, an oscilloscope (MDO3052, Tektronix, USA) and low-noise current preamplifier (SR570, Stanford Research Systems, USA) were connected to a Tektronix MDO3012 oscilloscope.

## Results and discussion

By considering motions of practical environmental wasting mechanical sources, it is crucial to design a multi-axial system to produce the high performance from TENGs. The components of the multi-axial system for a CTENG are shown in Figure 1(a) and S1, primarily consisting of fixed and moveable parts. As explained in methods, the fixed parts are the top layer, side bar, and bottom layer. The moving part, the Al molded middle layer, is freely vibrating in the x-axis direction. However, its motion is controlled between the top and bottom layers in the z-axis direction by adjusting the groove gaps *d*_G_ (0, 1, 2, and 2.5 mm). The bottom layer, containing the seven rectangular fins, was negatively charged due to PTFE films. The middle Al layer (with seven rectangular holes) was positively charged. Their contact areas are 8.4 cm^2^ in the x-axis direction and 4.56 cm^2^ in the z-axis direction, respectively (Supplementary Figure S2).

The working mechanism of the CTENG is shown in Figure 1(b,c). In this device, electrical power is obtained by two types of contact modes in x-axis and z-axis directions. First, for movement in the x-axis direction, contact is made between the fins and holes, as shown in Figure 1(b). When the PTFE surface at fins begins to contact the insides of Al holes, electrons are injected from the Al to the PTFE due to differences in their positions in the triboelectric series, as shown in Figure 1(b-i). When the Al begins to lose contact with the PTFE, electrons flow from the electrode bottom layer to the Al holes in the middle layer to maintain electrical neutrality, as shown in Figure 1(b-ii). Subsequently, when the alternate PTFE side surface begins to contact the Al holes, electrons within the Al holes begin to flow from the middle layer to the bottom layer, as shown in Figure 1(b-iii). Similarly, for movement in the z-direction, electrical power is generated by repeating the contact and release between the vibrating Al middle layer and top/bottom PTFE layers, as shown in Figure 1(c).

To investigate the influence of movable groove gap (*d*_G_) at the side parts on the x-axis and z-axis motion, a vibration frequency sweep experiment of both the x-axis and z-axis was undertaken, from 0 to 40 Hz, as shown in Figure 1(d–g). For vibration along the x-axis, the 2.5 mm gap provided the highest output voltage below 20 Hz. This result occurs because in the case of the other gaps, some input energy was converted to friction or crash energy between the middle layer and the sides. In other words, it could be confirmed that x-axis motion of the CTENG was restricted in the z-direction motion by the groove gaps of the size parts. Also, for vibration along the z-axis, increasing the groove gap (*d*_G_) led to an increase in the output performance because the movable distance of the middle vibrating layer increased, allowing contact with the bottom layer. It was thus found that the CTENG with a 2.5 mm gap is the most suitable for harvesting multi-directional energy.

**Figure 2.**
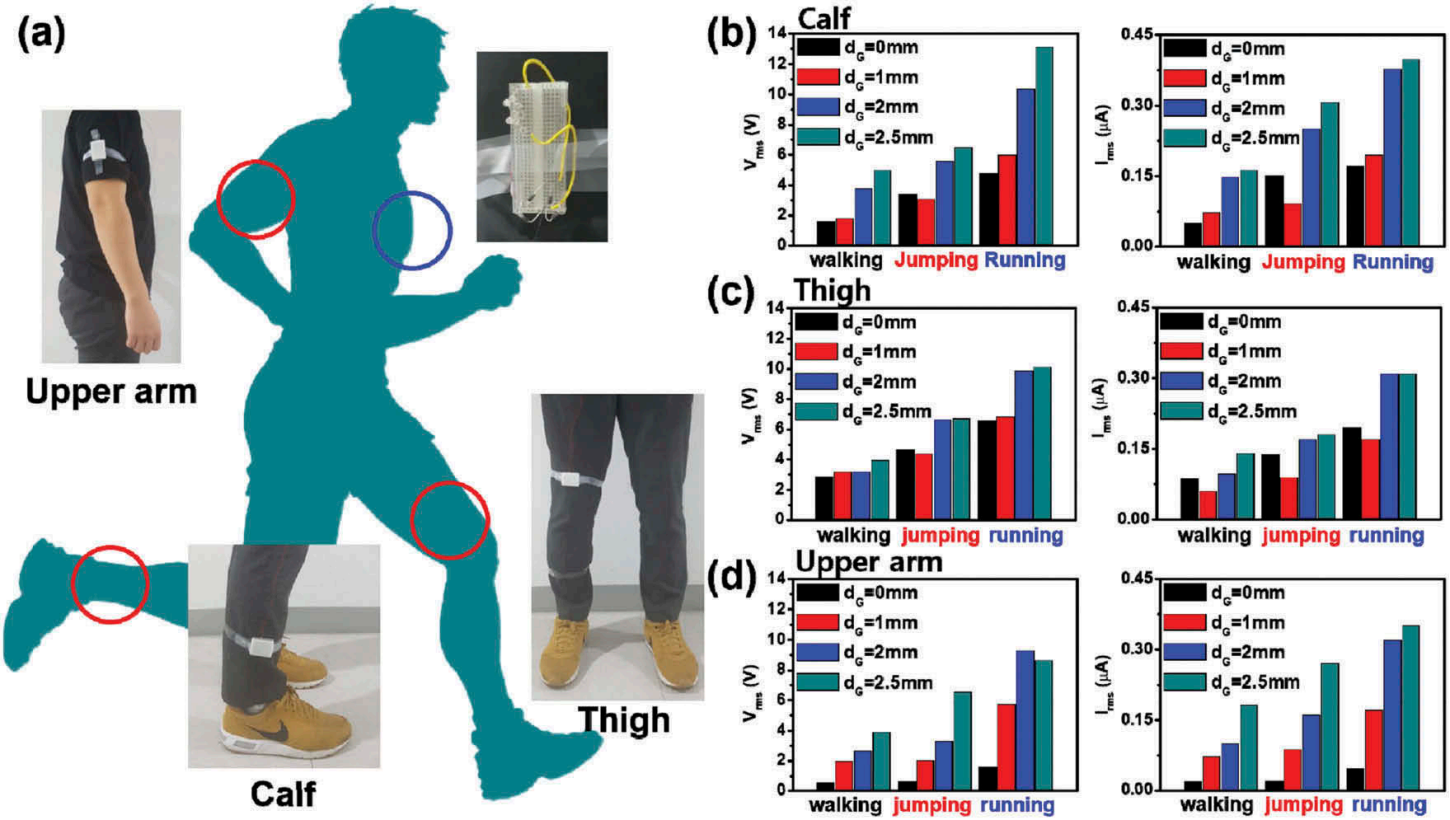
Output performance of the CTENG during three human motions: walking, jumping, and running (~5 m/s). (a) The illustrated position of the CTENG and 10 LEDs on the human body, including the calf, thigh, and upper arm. The dependency of electrical output of d_G_ 0, 1, 2 and 2.5 mm. (b-d) The RMS output voltage for 1 s about three motions and gap sizes, and pillar on the (b) calf, (c) thigh, and (d) upper arm.

To clearly understand the effects of multi-directional operation of a CTENG, we attached it to multiple points on the human body, including the upper arm, thigh, and calf, as shown in Figure 2(a). This figure shows the position of the CTENG with red circles, while blue circles give the position of the rectifying circuit and 10 light emitting diodes (LEDs). We measured the root mean square (RMS) output voltage and current while walking, jumping, and running (~5 m/s), as shown in Figure 2(b–d) and confirmed that the output voltage and current generated by the CTENG placed on the calf were highest, compared to output generated from placement on the thigh and upper arm. Figure 2(b–d) show the RMS output voltage and current generated by a 0, 1, 2, and 2.5 mm groove gap (*d*_G_), for the CTENG placed on the calf, thigh, and upper arm. We confirmed that the 2.5 mm gap generally provided higher contact times as well as higher output voltages and currents than the other gaps, as shown in Figures S3-S5. As a result, when placed on the calf, the RMS output voltage and current using a 2.5 mm gap were 13.12 V and 0.398 μA, respectively, while running (5 m/s). This result was approximately 2.7 times higher than results (4.74 V, and 0.17 μA) of the CTENG with *d*_G_ = 0 mm, as shown in Figure 2(b). Although the RMS output voltage and current on thigh and upper arm are almost the same, we explain the reason in Figure S6. Utilizing a 2.5 mm gap CTENG on the human body is effective due to the comb structure, because human motion is not confined to a single direction but rather consists of multi-directional movements. Furthermore, we verified that the CTENG when placed on the calf, thigh, and upper arm is capable of providing power to the rectifying circuits and nine LEDs when walking, jumping, and running (Supplementary Movies S1-S2).

**Figure 3.**
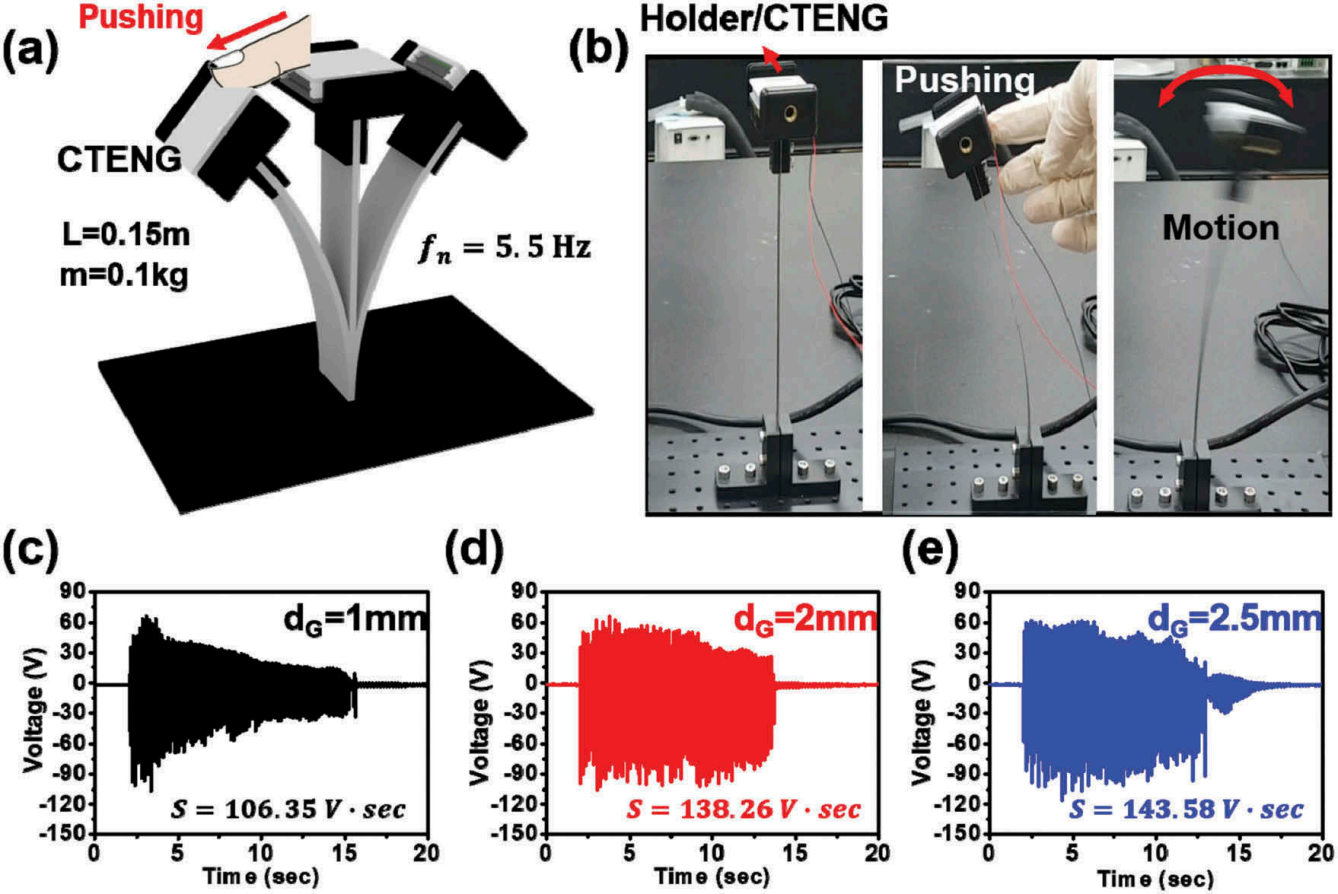
Hand-held pendulum (HHP) CTENG system and its mechanism (a) The schematic illustration with CTENG with 0.15 m length, 0.1 kg movable mass, and 5.5 Hz natural frequency and (b) the working of pendulum CTENG. The output voltage and natural frequency of the HHP system with CTENG of d_G_ (c) 1, (d) 2, and (e) 2.5 mm.

In order to practically operate a CTENG, we integrated a CTENG with HHP system, as shown in Figure 3(a). The system simply consists of a fixed bar, a CTENG holder, and the CTENG. The stainless bar has dimensions of 1 × 15 × 0.1 cm (a width, a length, and a thickness) and a Young’s modulus of 200 GPa. The holder (MPH 100, Pixmall, Korea) is used to affix the CTENG to the bar. A human hand could be simply used to provide a constant displacement by slightly pushing the HHP system and allow the HHP to continuously operate with a pendulum movement after release. The natural frequency of the HHP is facilely controlled by stiffness related to beam dimensions and the mass of the holder and CTENG. The natural frequency and stiffness of the pendulum is represented by relation (1) and (2), and is shown in Figure S7,

(1) $$f=\frac{1}{2\pi }\sqrt{\frac{k}{m}}$$

(2) $$k=\frac{3EI}{L^{3}}$$

where *f* is the natural frequency, *k* is the system stiffness, *m* is the movable mass, *E* is the Young’s modulus of the beam, *I* is the moment of inertia for the beam, and *L* is the beam length. Based on relations (1) and (2), a natural frequency of 5.6 Hz was calculated [24].

Figure 3(c–e) shows the output voltages at movable groove gaps (*d*_G_) of 1, 2, and 2.5 mm for the CTENG on HHP when the displacement was 4 cm and the movable mass was 0.1 kg. Based on relations (1) and (2), the stiffness of the system is 122.07 N/m and the theoretical natural frequency is 5.6 Hz, as shown in Figure S7. Similar to the theoretical value, the experimental natural frequency was 5.5 Hz. Although the frequency was similar, the output voltage varied as a function of movable gap size (*d*_G_) and the number of rectangular fins (1, 4, and 7), as shown in Figure 3(c–e) and Figure S8. For the 1, 2, and 2.5 mm groove gaps, the maximum output voltages were 106, 105, and 116 V, respectively. To accurately analyze total energy production in each condition, we calculated and compared the area of the output voltage with respect to the operation time. The CTENG on HHP, at a 2.5 mm groove gap, had the maximum area which was about 135% for 1 mm and about 104% for 2 mm, respectively. As expected, because the CTENG with a 2.5 mm groove gap can be well operated in x-axis direction as well as z-axis direction, the power generation on HHP system was also much effective and was the highest output power, compared with the CTENG with the restricted groove gaps (i.e. 1 and 2 mm).

**Figure 4.**
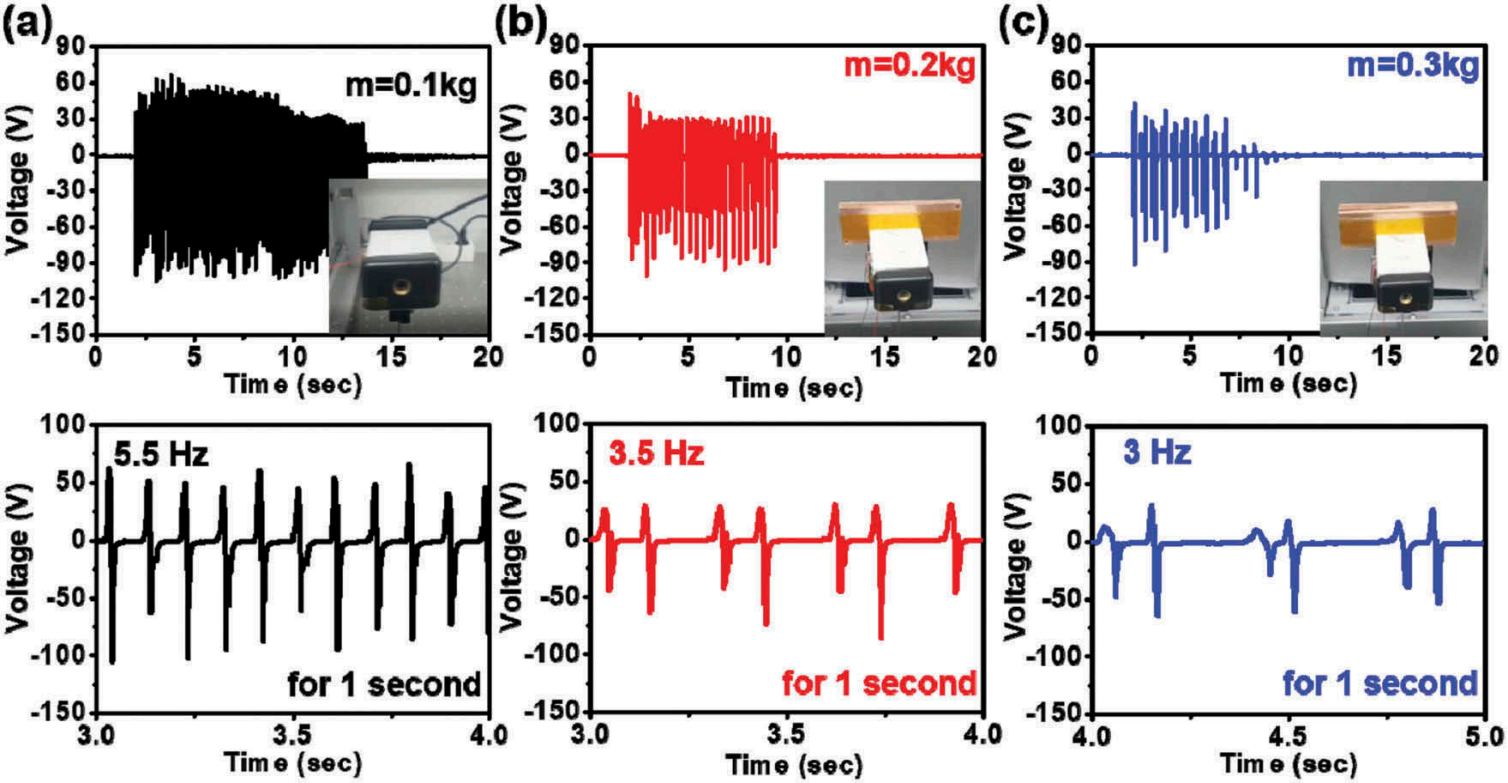
Dependence of the electrical outputs and operation times on movable mass. The measured voltage, current, and natural frequencies of an HHP system with CTENG under 4 cm of displacement and varying movable holder mass conditions, (a) 0.1, (b) 0.2, and (c) 0.3 kg. The inset contains an image of the movable mass and below graphs show a natural frequency for 1 s.

As mentioned previously, in relation (1), an increasing mass decreases the HHP natural frequency. To observe the output performance as a function of mass, we set the movable mass (m) to 0.1 (black), 0.2 (red) and 0.3 (blue) kg, as shown in Figure S7(a). Based on relation (1), the theoretical natural frequencies of the HHP with 0.1, 0.2 and 0.3 kg movable masses are 5.6, 3.9, and 3.2, respectively. Figure 4(a–c) shows the output voltage and natural frequency as a function of mass. An increase in the movable mass from 0.1 to 0.3 kg leads to a decrease in the output voltage from 105 V to 92 V, natural frequency from 5.5 to 3 Hz, and operation time from 11.7 s to 8.2 s at 4 cm of displacement. These results occur because the input energy converted to kinetic energy is greater than the TENG output as the movable mass increases.

**Figure 5.**
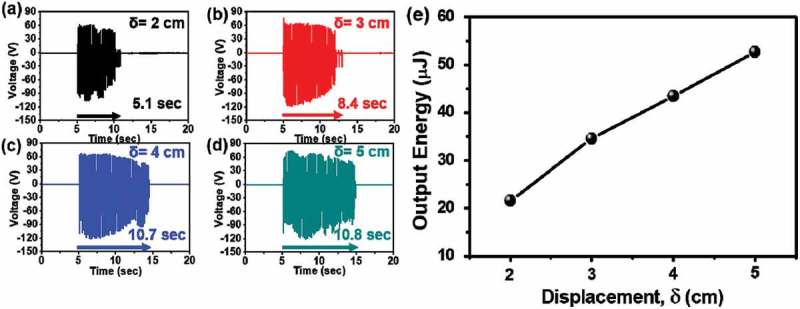
Dependence of the electrical outputs and operation time on the bending displacement of holder. The output voltages at (a) 2, (b) 3, (c) 4, and (d) 5 cm of bending displacement. (e) The output energy as a function of displacement of the HHP system with CTENG.

Figure 5 shows that increasing the displacement (2, 3, 4 and 5 cm) of the HHP increases the operation time (Δ*t*) and the output performance of the HHP because the input energy increases. But, over than 4 cm of the bending displacement (δ) of the HHP, the operation time is almost same because bar of HHP is excessive bending. The input energy of the HHP can be expressed as

(3) $$E_{input}=\frac{9EI}{2L^{3}}\delta^{2}$$

where E_input_ is the input energy and δ is the bending displacement of the HHP, as shown in Figure S7. Based on relation (3), the input energy increases with displacement and as a result, the maximum output performance (79 V and 14.43 μA) and operation time (10.8 s) are the highest at a displacement of 5 cm. Figure 5(a,b) compares operation times as a function of displacement. Also, Figure 5(a–d) shows the output voltage with increasing displacement, respectively. While the output performance at 5 cm displacement is greatest, a displacement of 4 cm produces results similar to 5 cm, as shown in Figure 5(a–d). We calculated the output energy (J) of the CTENG as a function of displacement based on relation (4),

(4) $$E_{output}=\int_{t_{2}}^{t_{1}}I^{2}R\;dt$$

where t_1_ and t_2_ are the start and end of the operation time, respectively, *I* is the output current, and R is the resistance of the CTENG circuit. E_output_ increased with an increased E_input_, from 21.6 μJ to 52.7 μJ, but, the Relative Efficiency was decreased as shown in Figure 5(e) and Figure S9.

**Figure 6.**
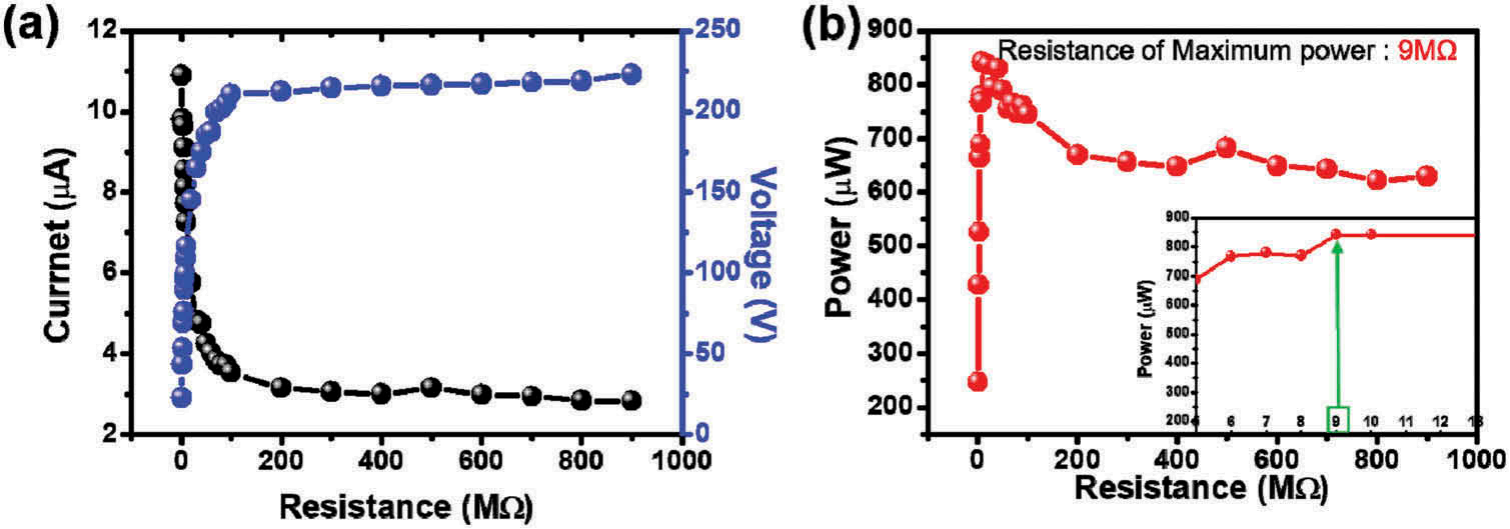
The electrical output performance of the HHP system with CTENG. The output (a) voltage, current, and (b) peak power of HHP system with CTENG with a load resistance varying from 1 to 900 MΩ.

Figure 6(a–b) shows the output voltage, current, and power with external loads varying from 1 to 100 MΩ under 0.1 kg of mass and 4 cm of displacement. The instantaneous power from the external resistance is 842 μW at a resistance of 9 MΩ. For the conventional TENGs, they have a maximum output power at a resistance between 1 an﻿d 100 MΩ because they have a high internal resistance in case of metal-to-polymer and polymer-polymer contact. When we compare our system with previous studies, we believe that our resistance at the maximum output power is reasonable [9,25–28].

**Figure 7.**
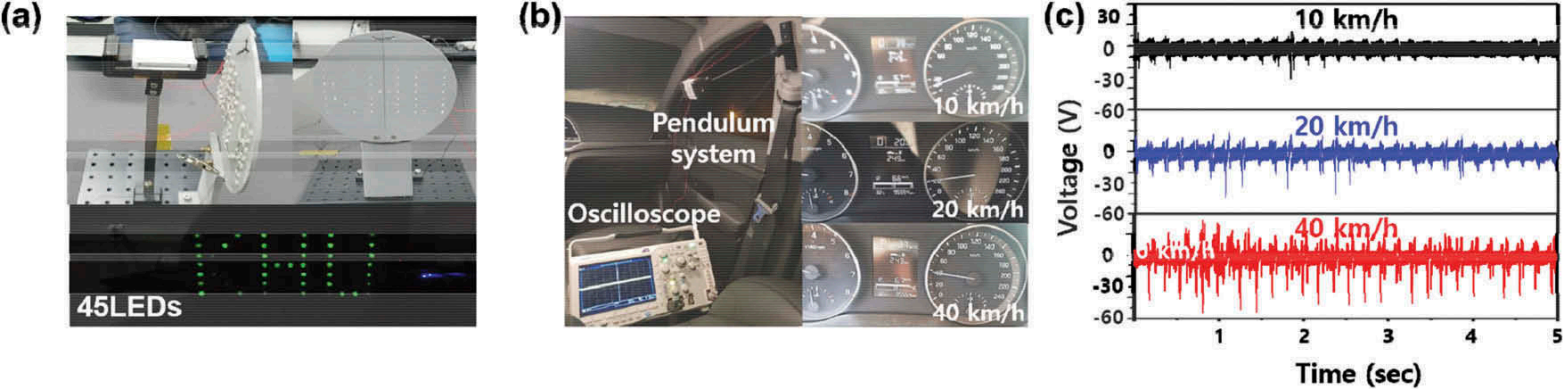
Application of the HHP system with CTENG. (a) Powering 45 serially connected green LEDs by the HHP system with CTENG when harvesting vehicle driving vibrations. (b) Vehicle traveling at speeds of 10, 20, and 30 km/h. (c) The output voltage results during vehicle driving.

To validate the capacity of the CTENG in practical applications, the HHP was connected to 45 LEDs and lighted capital letters ‘KHU’ LEDs under pendulum vibration by hand, as shown in Figure 7(a) (see Supplementary Movie S3). Additionally, to demonstrate the application of the HHP integrated CTENG system, it was placed on the right side of a car dashboard and experimental measurements were taken at driving speeds of 10, 20, and 40 km/h, as shown in Figure 7(b). Figure 7(c) shows that the output voltage increased with increasing vehicle velocity due to vibrations of the car dashboard, which were more likely to occur at low frequencies (near 5 Hz). This is similar to the natural frequency of a pendulum, thus demonstrating the possibility for a self-powered velocity monitoring system. (see Supplementary Movie S4)

## Conclusions

In this study, we have successfully demonstrated a CTENG capable of effectively scavenging multi-directional motions from human movements such as walking, jumping, and running, and temporary continuous operation via a HHP system. Firstly, to effectively harvesting multi-directional human movements, we designed a comb-structure based TENG and movable groove displacement (*d*_G_) of side parts. While running (~5 m/s), we could obtain RMS power value of 5.28 μW (i.e. 13.12 V and 0.4 μA) for 1 s by attaching CTENG at *d*_G_ = 2.5 mm to a person’s calf and turn on 10 LEDs. With these results, we integrated a CTENG with a HHP system with a natural frequency of 5.5 Hz. The natural frequency and input energy of our HHP system can be easily controlled by changing the holder mass and initial bending displacement, thus producing different output behaviors for the CTENG. Under the optimal HHP-based CTENG system design, the maximum output reaches 116 V at 6.5 μA under 0.1 kg mass and 4 cm bending displacement conditions. The corresponding output energy is 52.7 μJ for an operation time of 10.8 s. The maximum instantaneous power was 842 μW at the resistance of 9 MΩ. Our HHP-CTENG system could power 45 LEDs and showed different output behaviors by varying the driving velocity of a vehicle, thus demonstrating the possibility for a self-powered velocity monitoring system. Finally, our multi-directional CTENG and HHP system are capable of generating electrical energy anywhere, anytime from any motions and can further serve as an approach to harvesting and monitoring mechanical motions and velocity for self-powered electronics and miniaturization of IoT devices.
