# Supplementary material for: Comb-structured triboelectric nanogenerators for multi-directional energy scavenging from human movements
Source: Sci Technol Adv Mater. 2019 Jul 1;20(1):725–32. doi: 10.1080/14686996.2019.1630856 (PMC6610512; doi:10.1080/14686996.2019.1630856)
Supplement: Supplemental Material [file TSTA_A_1630856_SM6211.zip › Suppl/supplementary_production.pdf]

Supplementary materials

Comb-structured TENGs for multi-directional energy scavenging and their operation via hand-handled pendulum system

Hee Jae Hwang[1], Yeonsuk Jeong[1], Kyungwho Choi[2], Dongseob Kim[3], Jinhyoung Park[4#], and Dukhyun Choi[1#]

[1] *Department of Mechanical Engineering, Kyung Hee University, Yongin 17104, Korea*
[2] *New Transportation Innovative Research Center, Korea Railroad Research Institute, Uiwang-si 16105, Korea*

[3] *Aircraft System Technology Group, Korea Institute of Industrail Technology (KITECH), 57, Yangho-gil, Yeongcheon-si, Gyeongbuk-do 38822, Korea*

[4] *Construction Equipment R&D Group, Korea Institute of Industrail Technology (KITECH), 288-1, Daehak-ri, Hayang-eup, Gyeongsan-si, Gyeongsangbuk-do 712091, Korea*

(# These authors were co-corresponding authors)

[#]Correspondence to dchoi@khu.ac.kr (D. Choi) and j.h.park@kitech.ac.kr

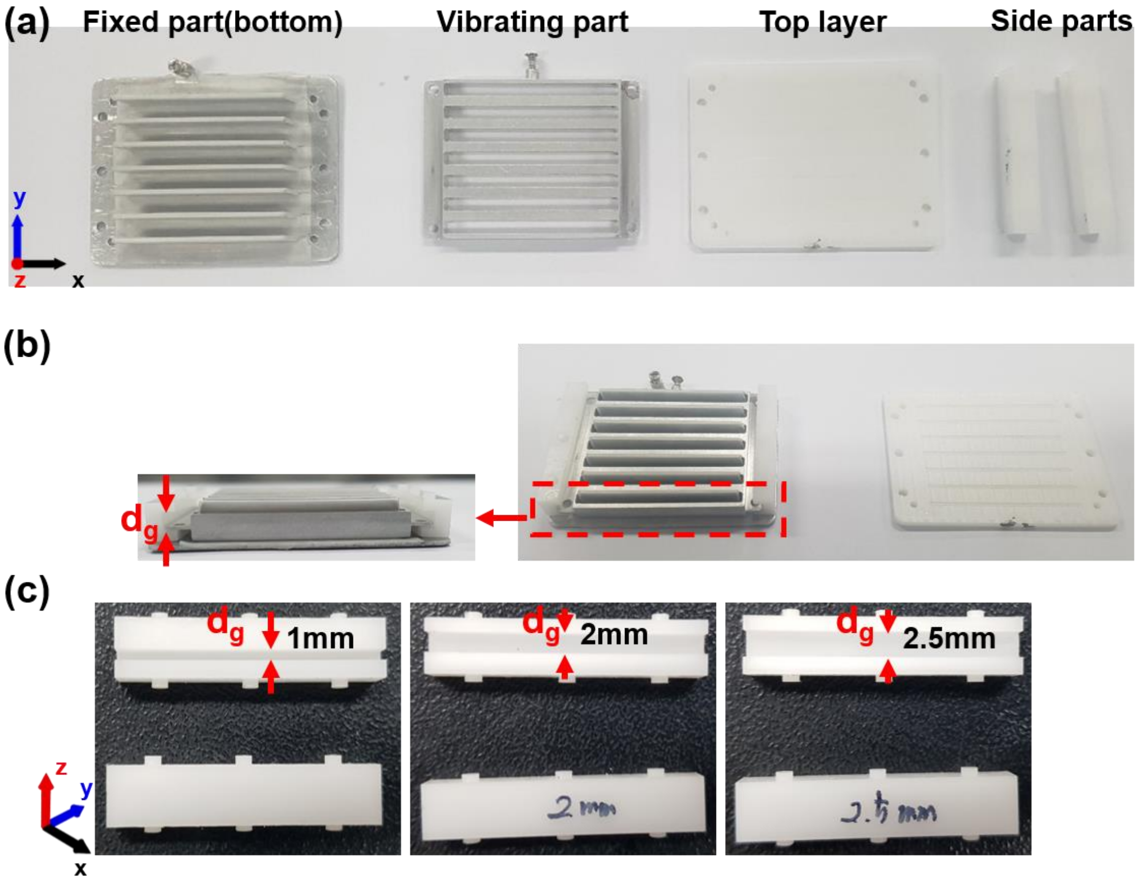

Supplementary Figure 1. The photographs components of multi-direction and comb-structured TENG. (a) The aluminium molds are fixed part (bottom) and vibrating part (middle). The Teflon molds are fixed part (up) and side parts (b) The combined device with fixed part, vibrating part and side parts (c) The gap size of d_G 1, 2, 2.5 mm of side parts.

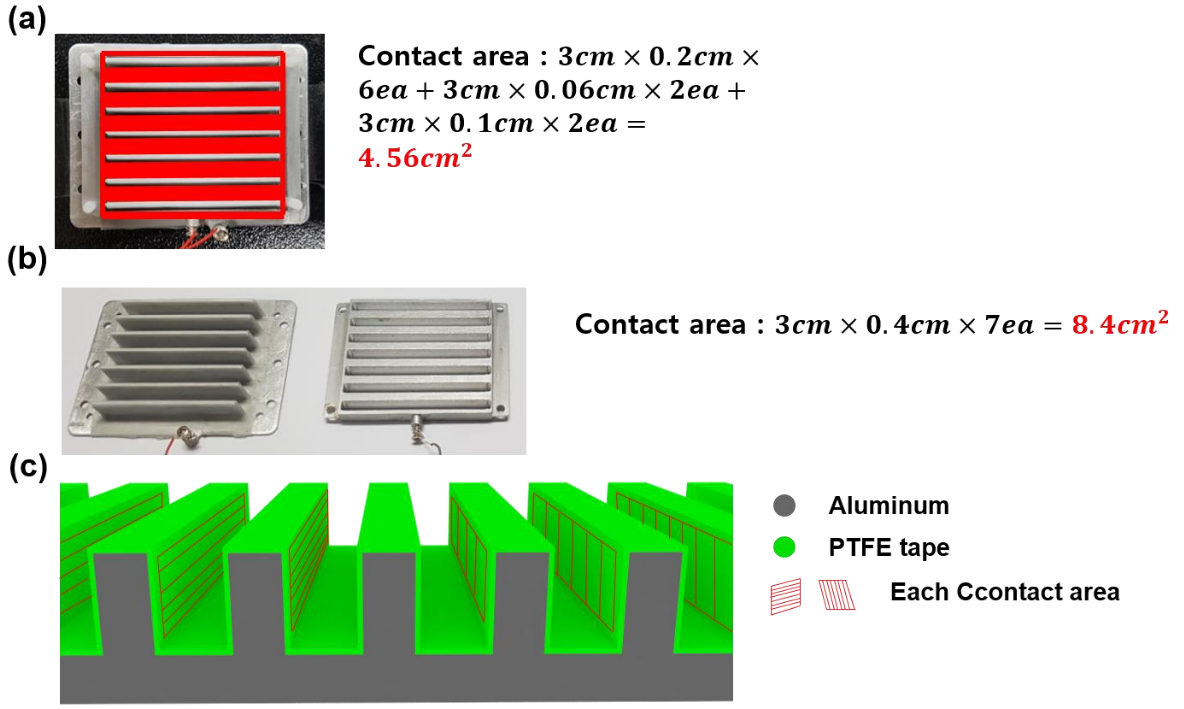

Supplementary Figure 2. Contact area with axis. The contact area of (a) z-axis and (b) x-axis. (c) The schematic of bottom layer contact area.

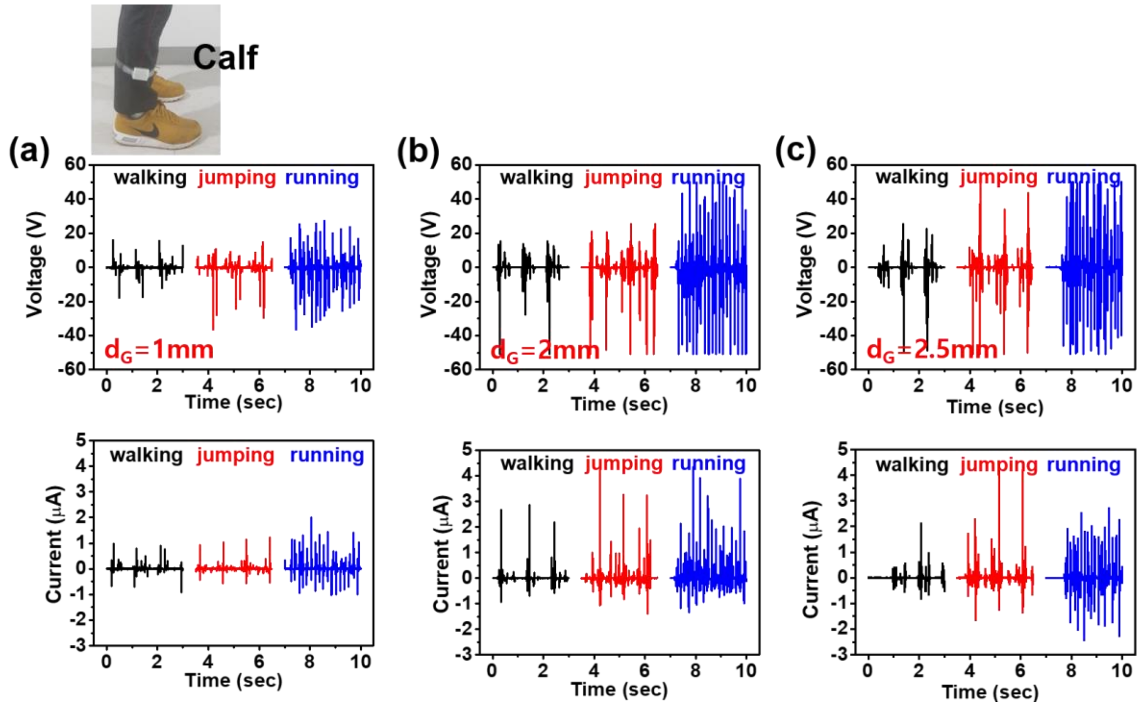

Supplementary Figure 3. The output performance of the CTENG on calf in three motion walking, jumping, running (~ 5 m/s). Dependency of electrical voltage and current of CTENG the gap size of holder, d_G (a) 1, (b) 2, (c) 2.5 mm on calf.

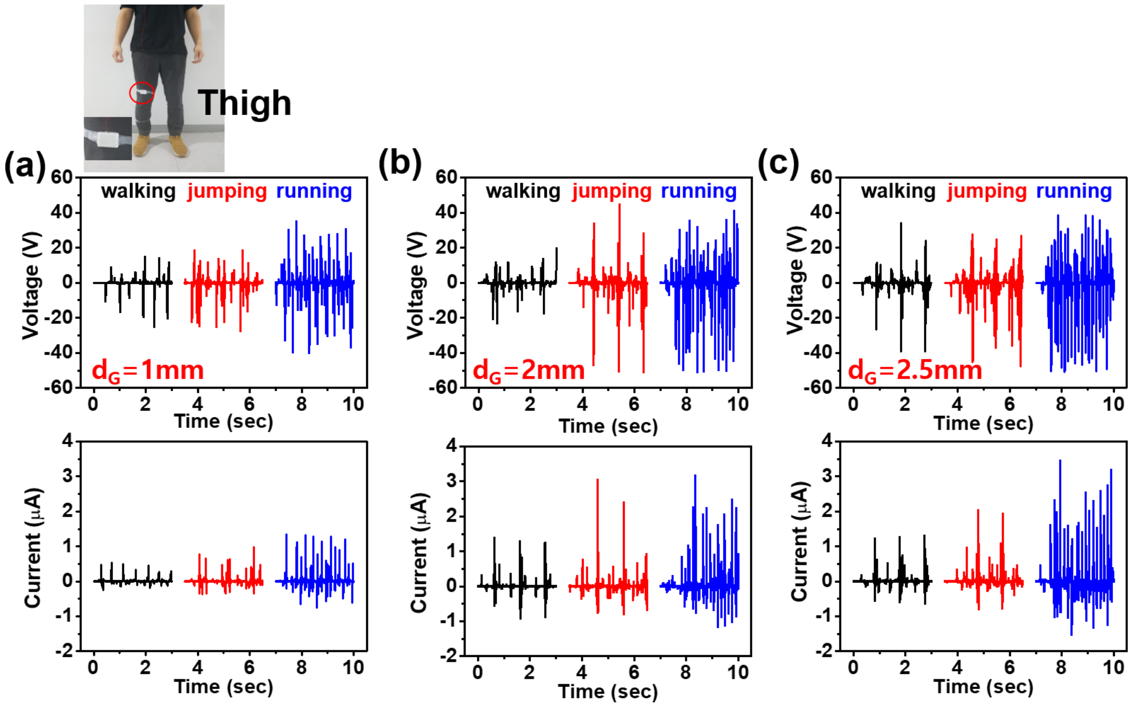

Supplementary Figure 4. The output performance of the CTENG on thigh in three motion, walking, jumping, running (~ 5 m/s). Dependency of electrical voltage and current of CTENG the gap size of holder, d_G (a) 1, (b) 2, (c) 2.5 mm on thigh.

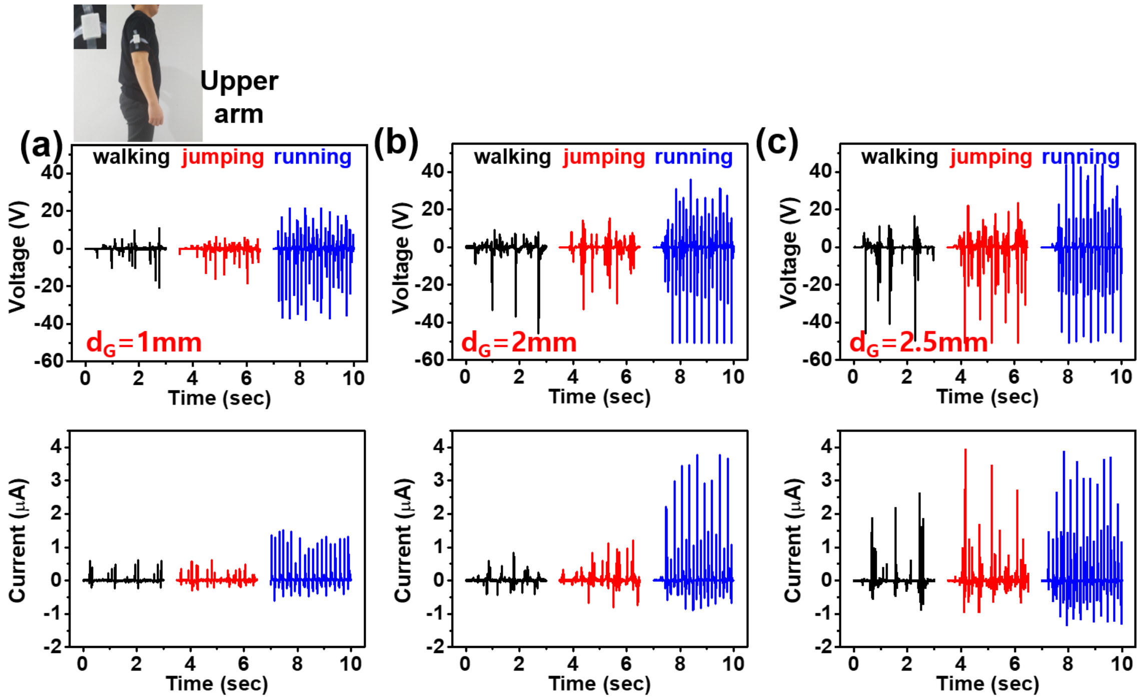

Supplementary Figure 5. The output performance of the CTENG on upper arm in three motion, walking, jumping, running (~ 5 m/s). Dependency of electrical voltage and current of CTENG the gap size of holder, d_G (a) 1, (b) 2, (c) 2.5 mm on upper arm.

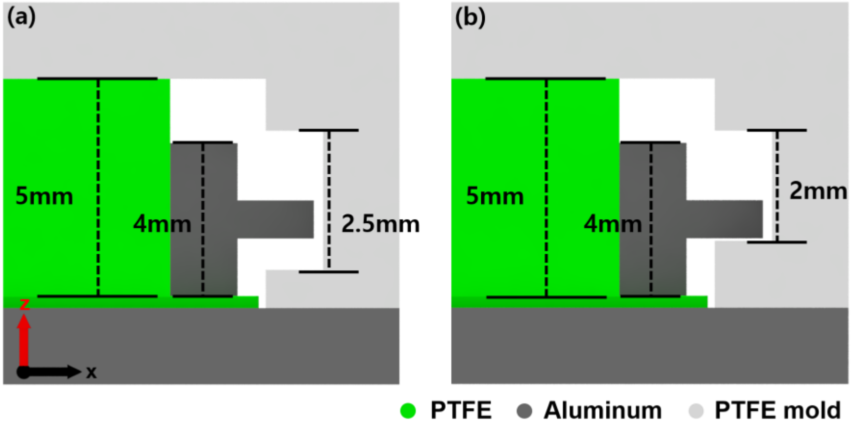

Supplementary Figure 6. The schematic cross section of CTENG with (a) d_G 2.5mm and (b)d_G 2mm. The movable length of middle layer as a vibrating part on z-axis is 5.5mm and the height of middle layer is 4mm as shown in Figure 1. The middle layer is almost attached to bottom layer in case of d_G 2.5mm and d_G 2mm. I estimate that this is the almost same reason to the output performance of CTENG (d_G 2.5mm) and d_G 2mm

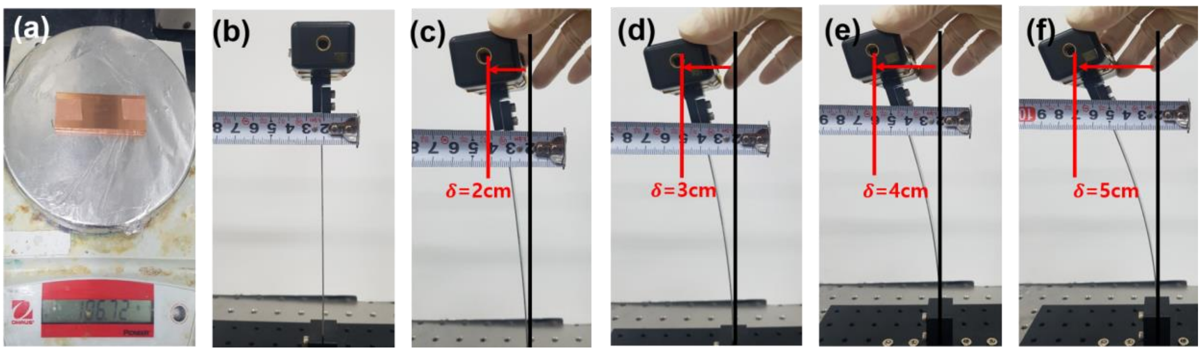

Supplementary Figure 7. The copper sheet to control movable mass and explanation of stiffness and natural frequency. (a) the copper sheet (b-f) The displacement of holder of pendulum system from 0 cm to 5 cm. Based on hook's law, the force (P) is presented like presented like relation (S1)

$$\mathrm{P} = \mathrm{k}\delta = \frac{3\mathrm{EI}}{L^3}\delta \tag{S1}$$

Where, k is the stiffness, δ is the displacement of movable mass, E is the Young's modulus, I is the inertia moment and L is the length of pendulum bar. [1] Therefore, the stiffness is presented like relation (S2)

$$\mathrm{k} = \frac{3\mathrm{EI}}{L^3} \tag{S2}$$

And then, natural frequency is presented like relation (S3) by relation (1) and (S2)

$$\mathrm{f} = \frac{1}{2\pi}\sqrt{\frac{3EI}{mL^3}} \tag{S3}$$

To calculate input energy, we used calculation of the moment (M) and strain energy (U_s) like relation (S4) and (S5)

$$\mathrm{M} = -\mathrm{PL} \tag{S4}$$

$$U_s = \int_0^L \frac{M^2}{2EI}dx \tag{S5}$$

By relation (S1), relation (S4) is presented like relation (S6) and the final input energy relation is presented like (S7) by relation (S6) and E, I, L, δ is constant,

$$\mathrm{M} = -\frac{3\mathrm{EI}}{L^2} \tag{S6}$$

$$U_s = \frac{9EI}{2L^3}\delta^2 \tag{S7}$$

[1]. Rao SS. Mechanical vibrations fifth edition. Prentice Hall;1995

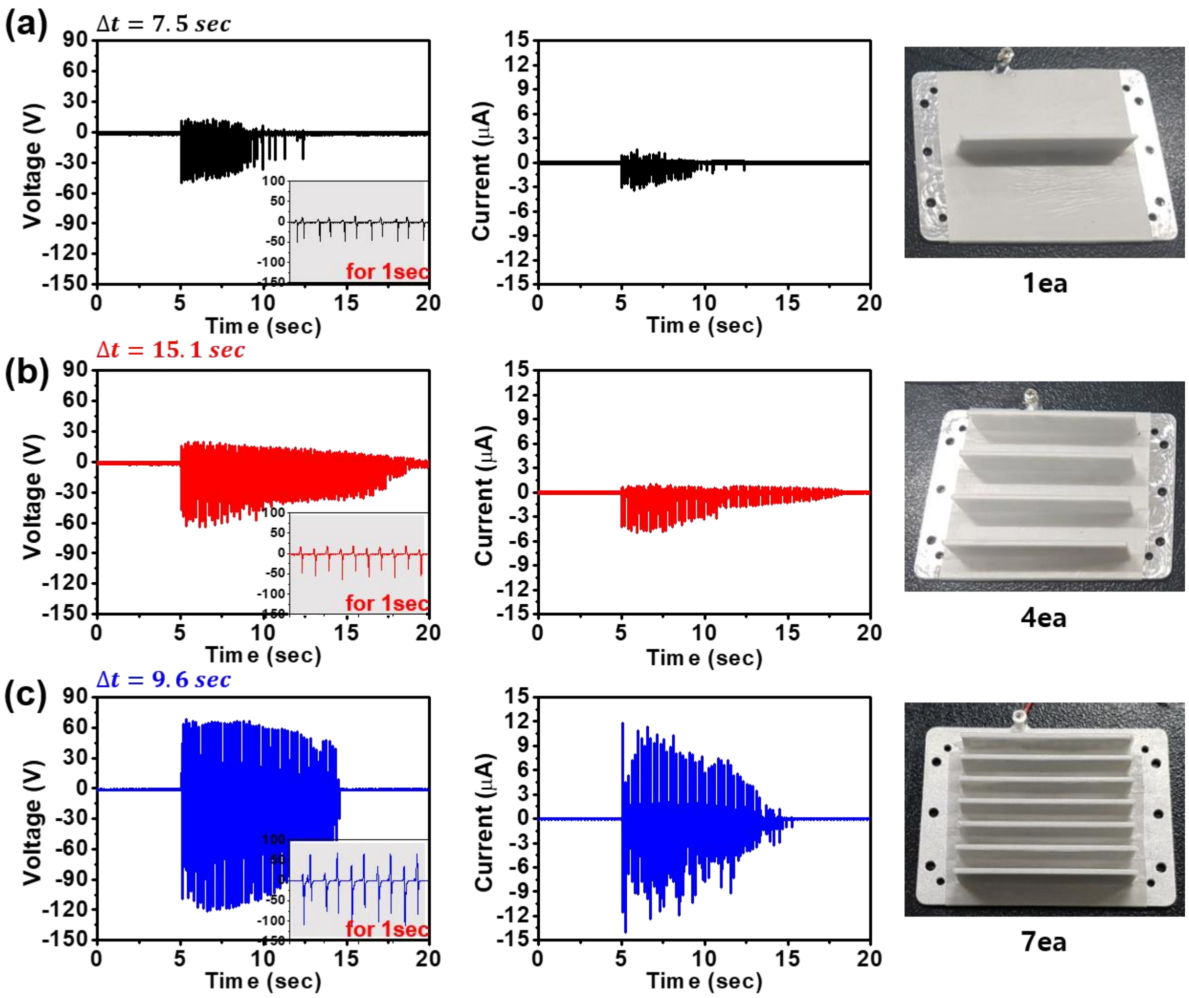

Supplementary Figure 8. The output voltage and current with counts of comb. (a) 1 ea, (b) 4 ea and (c) 7 ea fins.

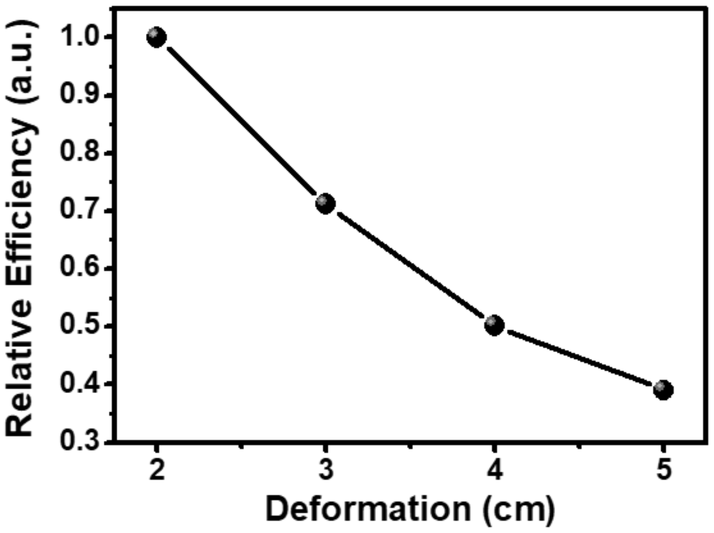

Supplementary Figure 9. The Relative Efficiency of HHP system with CTENG by increasing bending displacement.

We calculated the Relative Efficiency of HHP system with CTENG by using relation (S8) and (S9)

$$\eta = \frac{E_{output}}{E_{input}} \tag{S8}$$

$$RE_x = \frac{\eta_{xcm}}{\eta_{2cm}} \tag{S9}$$

where η is the efficiency of HHP system with CTENG, E_{output} is the electrical output energy of HHP system with CTENG, E_{input} is the input energy of HHP system by bending displacement, RE_x is the relative efficiency of HHP system with CTENG about η with 2cm (η_{2cm}) of bending displacement when bending displacement of HHP system is x cm and η_x is the efficiency of bending displacement x cm. We confirmed that the Relative Efficiency of hand-handled pendulum with CTENG was decreased by increasing bending displacement because energy loss like friction energy and crash energy are occurred while HHP system with CTENG are operating as shown in figure S9.
